# A review: targeting UBR5 domains to mediate emerging roles and mechanisms – chance or necessity?

**DOI:** 10.1097/JS9.0000000000001541

**Published:** 2024-05-03

**Authors:** Yizhu Wang, Kaiyi Niu, Yanlong Shi, Feilong Zhou, Xinhao Li, Yunxin Li, Tianyi Chen, Yewei Zhang

**Affiliations:** Hepatopancreatobiliary Center, The Second Affiliated Hospital of Nanjing Medical University, Nanjing, Jiangsu Province, People’s Republic of China

**Keywords:** diseases, E3 ligase, pathophysiological function, structure, UBR5

## Abstract

Ubiquitinases are known to catalyze ubiquitin chains on target proteins to regulate various physiological functions like cell proliferation, autophagy, apoptosis, and cell cycle progression. As a member of E3 ligase, ubiquitin protein ligase E3 component n-recognin 5 (UBR5) belongs to the HECT E3 ligase and has been reported to be correlated with various pathophysiological processes. In this review, the authors give a comprehensive insight into the structure and function of UBR5. The authors discuss the specific domains of UBR5 and explore their biological functions separately. Furthermore, the authors describe the involvement of UBR5 in different pathophysiological conditions, including immune response, virus infection, DNA damage response, and protein quality control. Moreover, the authors provide a thorough summary of the important roles and regulatory mechanisms of UBR5 in cancers and other diseases. On the whole, investigating the domains and functions of UBR5, elucidating the underlying mechanisms of UBR5 with various substrates in detail may provide new theoretical basis for the treatment of diseases, including cancers, which could improve future studies to construct novel UBR5-targeted therapy strategies.

## Introduction

Highlights

- We give a comprehensive insight into the structure and function of UBR5.
- We discuss the specific domains of UBR5 and explore their biological functions separately.
- We describe the involvement of UBR5 in different pathophysiological conditions, including immune response, virus infection, DNA damage response, and protein quality control.
- We provide a thorough summary of the important roles and regulatory mechanisms of UBR5 in cancers and other diseases.

**Figure 1:**
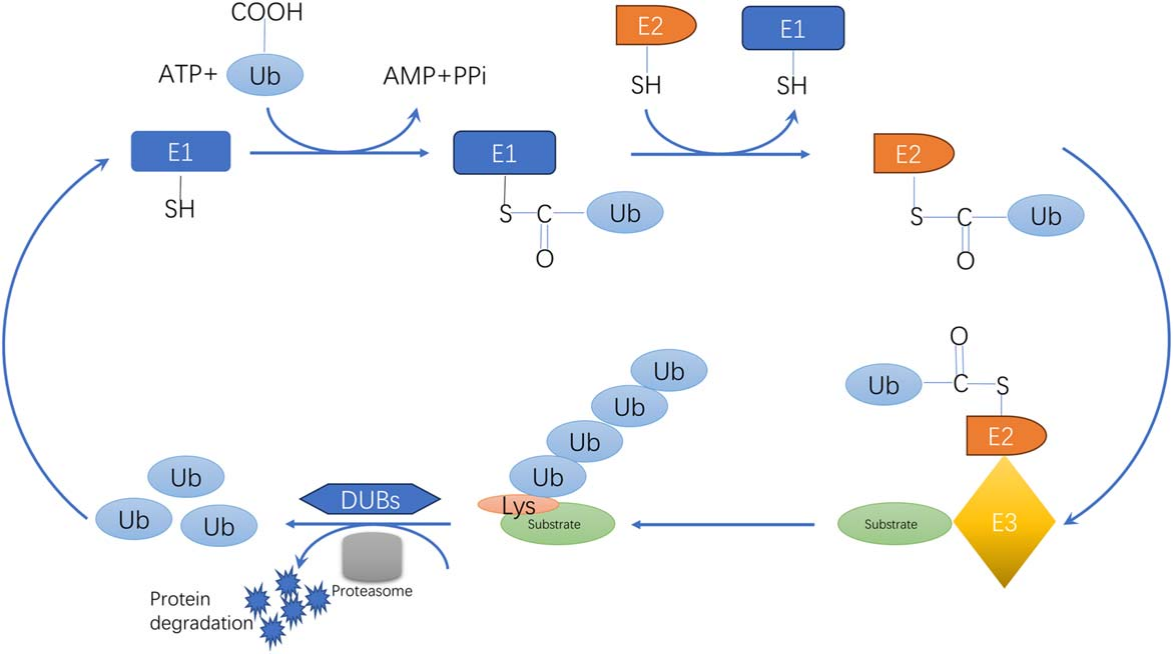
Ubiquitination is accomplished by a series of interdependent enzymatic reactions: ubiquitin activated by E1 is transferred to E2, and E3 is responsible for the recruitment of substrate proteins. DUBs remove ubiquitin from the substrate.

Proteins are the most important performers of vital activities. It was proper protein degradation that is essential to maintain function and homeostasis in organisms^1,2^. The proteasomal and lysosomal pathways are the major protein degradation pathways, which is responsible for cellular processes like cell cycle, signaling, apoptosis, and autophagy^3,4^. Ubiquitin (Ub), a small-molecule protein of 76 amino acid residues, was initially identified as the 26S proteasome trigger for protein degradation in eukaryotic cells, and the ubiquitin proteasome system (UPS) pathway targets the degradation of proteins labeled by Ub selectively^5^. Generally, this process occurs through the sequential action of Ub-activating enzymes (E1), Ub-conjugating enzymes (E2), and Ub ligases (E3). Especially, the core of ubiquitination modification is the formation of a stable isopeptide bond between the glycine of ubiquitin and the lysine residue of the target protein. The E3 enzymes recognize and differentiate the specificity of various substrates, and are hence thought to play the most significant role in ubiquitination^6,7^. For example, RNF216 promotes Beclin-1 proteasomal degradation to inhibit autophagy by assembling K48-linked Ub chains on Beclin-1^8^. MAGI3, a novel E3 ligase, degrades c-Myc in colorectal cancer (CRC) and modulates chemosensitivity in CRC^9^. In addition, polyubiquitination is also dependent on E4 ligases, which are considered Ub chain extension factors and involved in the formation of E3-E4 or E4-substrate complexes that extend Ub chains assembled by E1, E2, and E3 enzymes^10,11^ (Fig. 1).

As estimated, the human genome encodes over 600 E3 ligases. Depending on the structural domains of the E3 ligases and their Ub transfer mechanisms, they are classified into three main categories: RING (Really Interesting New Gene), HECT (Homologous to E6AP C-Terminus), and RBR (RING-between-RING)^12^. RING E3 is the largest of the three E3 categories with ~600 members that has characteristic RING structural domain, which is required for recruitment of the E2-Ub complex and stimulation of Ub transfer^13^. Due to the lack of an active cysteine (Cys) site, RING E3 directly transfers Ub from the E2-Ub intermediate to the substrate^14^. In contrast, RBR E3 and HECT contain catalytic Cys that first receive Ub from the E2-Ub complex, form the E3-Ub thioester intermediate, and then transfer Ub to the substrates^15^. The RBR E3 ligases have 14 members and typically contain two ring structural domains (ring 1 and 2) and an in-between RING (IBR) zinc-binding region^16^. HECT E3, on the other hand, contains 28 members and is characterized by the presence of various substrate binding N-terminal region (NTR) and a conserved C-terminal HECT domain of ~350 amino acids^17^. HECT E3 is subdivided into three groups based on their N-terminal protein-protein interaction structural domains: firstly, the NEDD4 family, which is characterized by the presence of WW structural domains that bind the PY motifs of substrates; secondly, the HERC family that contains regulator of chromatin condensation-1 (RCC1)-like domains (RLDs); and finally, the HECT family with protein-protein interaction domains^18^. The conserved C-terminal HECT domain of HECT has a bilobed structure consisting of a larger N-terminal lobe (N-lobe) and a smaller C-terminal lobe (C-lobe) tethered to each other by a short flexible hinge loop. The N-lobe provides a docking site for the E2 enzymes, while the C-lobe contains active Cys residues. The flexible hinge allows different rearrangements of the two leaflets, which is essential for HECT-mediated catalysis^19,20^.

**Figure 2:**
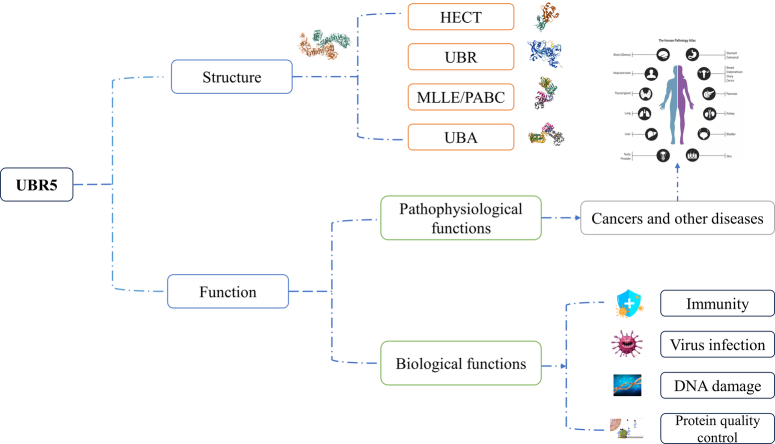
The overall framework of this study.

There exists a unique class of E3 Ub ligases that structurally contain a conserved substrate-recognition structural domain known as the UBR box (70-residue zinc finger-type UBR-box domain) and its member includes UBR1-UBR7^21^. UBRs recognize the N-degron of various short-lived and misfolded proteins in cell cytoplasm or nucleus, regulate protein degradation homeostasis, and thus maintain the quality control of protein metabolism, which is known as the law of the N-terminus^22^. UBRs have various functions and properties. For example, UBR4 has been reported to be involved in cell surface protein turnover and to play a role in the homeostasis of cell surface proteins. Deletion of UBR4 induces depletion of many proteins in the plasma membrane, such as EGFR and PDGFR-β^23^. UBR5 not only regulates genome stability, but also modulates transcription factors in a c-Myc-centered network to control gene expression^24,25^. In addition, it has been reported that UBR7 acts as a tumor suppressor in hepatocellular carcinoma (HCC) by targeting the Keap1/Nrf2/Bach1/HK2 axis and aerobic glycolysis^26^. UBR5 is categorized in the HECT E3 family due to its HECT structural domain. UBR5, as an integral member of the UBR family, plays important roles in viral infection, DNA damage response (DDR), protein degradation, cell proliferation and apoptosis, tumor immunity, and drug resistance. Furthermore, UBR5 regulates the AKT-mTOR signaling pathway in lung cancer and promotes lung carcinogenesis^27^. Despite the large number of studies on the structure and function of UBR to date, there is a lack of detailed and specific reviews of the structure and functions of UBR5. Therefore, in this review, we provide a detailed structural description of UBR5, summarize the roles of UBR5 dependent or independent of Ub ligase activity, systematically elaborate the biological functions of UBR5 in diseases, and shed new insights into the mechanism of UBR5 as a clinical therapeutic target (Fig. 2).

### Structure of UBR5

A total of seven UBR cassette E3 ligases (UBR 1-7) have been identified in mammals, and they have both structural similarities and differences. All UBR proteins recognize N-degrons via the UBR box, which is the hallmark of the UBR family. In addition, different UBR proteins also possess special domains for E2 binding or Ub conjugation. For example, UBR1-3 has a RING domain, which belongs to the RING family. UBR7 has a PHD domain, a Cys and His residue pattern similar to the RING domain, and proteins with this domain are mainly involved in gene regulation in the nucleus^28,29^.

**Figure 3:**
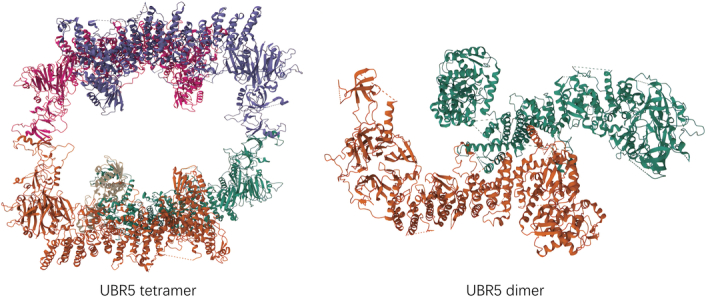
The crystal structure of UBR5 dimer and tetramer.

UBR5, a nuclear phosphoprotein that localized to human chromosome 8q22.3, was originally identified as a homolog of the tumor suppressor gene hyperplastic discs in Drosophila^30^. The central feature of UBR5 is its large α-solenoid helical scaffold, decorated with several putative protein-binding domains that facilitate E2-Ub transfer, oligomerization, and catalysis^31^. Analysis of electron‐cryo microscopy (cryo‐EM) showed that UBR5 monomers were crescent-shaped, and UBR5 oligomers were dimer and tetramer. The formation of UBR5 tetramer was mediated by intermolecular small β barrel domain2 (SBB2-SBB2) interaction between two UBR5 dimers. Since the tetramer interface is much smaller than the dimer interface, the tetramer may be less stable than the dimer^32^. However, oligomerization is not necessary for enzyme function and may help to locate each protein-binding module near the catalytic HECT domain, rather than directly mediating sustained synthesis capacity. Like other HECT E3s, the HECT domain of UBR5 is located at the C-terminal and has a larger N-lobe interacting with E2 and a C-lobe containing catalytic Cys^33^. In addition to HECT, UBR5 contains a Ub association domain (UBA) that interacts with Ub^34^, two predicted small β barrel domains (SBB1 and SBB2), a 70-residue zinc-finger UBR-Box^35^ that recognizes N-degron, and a MLLE domain that mediates protein-protein interactions^36^. These domains are fundamental to the normal functioning of UBR5. In the following sections, we discuss the characteristics and functions of various structural domains of UBR5 minutely (Fig. 3).

### HECT domain

The HECT domain of UBR5 has a bilobed structure: the larger N-terminal ‘N-lobe’ binds E2s, and the smaller C-terminal ‘C-lobe’ contains catalytic Cys, which are tethered to each other by a short and flexible linker. The two common N-lobe and C-lobe configurations of HECT are ‘L’ and “inverted ‘T’. They differ by rotating ≈150° around the flexible linker, placing the C-lobe on one side (L) or toward the middle of the N-lobe (inverted T). HECT E3s in the ‘inverted T’ conformation receive Ub and transfer Ub to the substrate in the ‘L’ conformation^37–39^. HECT E3s-catalyzed Ub transfer proceeds in two steps: the first step is a trans-thioesterification reaction in which the E3 enzymes take over Ub from the Ub-loaded E2 enzymes and form a thioester bond between its catalytic Cys residue and the C-terminus of Ub. In the second step, the activated C-terminus of Ub is nucleophilic attacked by the primary amino group of the target protein, resulting in an isopeptide bond between Ub and the target protein. This is accompanied by a rotation between the N and C lobes^40^. In addition, it has been shown that there is a special region called plug loop in the UBR5 HECT domain, which blocks the space between the N and C lobes. This loop does not lie before the hinge or HECT domain in linear sequence but is separated by nearly 200 unstructured residues. However, the deletion mutant of the plug loop still rapidly formed Ub chains, indicating that it is not essential for the intrinsic activity of UBR5. Deletion mutants of the plug loop are unstable over time, a feature that could serve as a folding aid for the HECT domain during protein assembly^31^. Besides, in the UBR5 dimer, the NTR of one subunit is close to the HECT domain of the other subunit, forming a gap named ‘intermolecular E2-Ub jaw’, which was speculated to bind an E2-Ub^32^.

The HECT domain plays a central role in the function of UBR5. Studies have shown that the loss of the HECT domain can lead to the arrest of maturation of B cells and follicular B cells, and Follicular B cells have abnormal phenotypes, including low expression of immunoglobulin D (IgD) and high expression of IgM. After immune stimulation, B cells without the HECT domain show reduced germinal center formation and decreased antibody-producing plasma cells, suggesting a functional defect^41^. In addition, UBR5 has been reported to play an antagonistic role in MERS-CoV pathogenesis by attenuating the immune escape of MERS-CoV via promoting ubiquitination and degradation of ORF4b. Moreover, only UBR5 containing the HECT domain can interact with ORF4b^42^. It has also been found that PEPCK1 (phosphoenolpyruvate carboxykinase) is acetylated by P300 acetyltransferase. This acetylation stimulates the interaction between PEPCK1 and UBR5, thereby promoting PEPCK1 ubiquitination and degradation. Also, the C-terminal HECT domain of UBR5 is required for this effect. Exogenous expression of the UBR5 HECT deletion mutant is unable to reduce PEPCK1 protein levels, suggesting that UBR5 HECT-mediated E3 ligase activity is required for UBR5 to reduce PEPCK1 protein levels^43^.

### UBR domain

According to the N-terminal rule, the protein lifetime depends on the characteristics of its N-terminal residues called N-degrons. Substrates of the N-terminal regular pathway are cytosolic short-lived proteins with type 1 (Arg, Lys, and His; Positively charged) and type 2 (Phe, Tyr, Trp, Leu, and Ile) N-degrons. N-degrons can be exposed by N-terminal methionine (Met) excision or by proteolytic cleavage of other stable proteins^44–47^. The substrates with type 1 N-degrons contain an N-terminal basic residue that can be recognized by the UBR box domain of the E3 Ub ligase UBR1. In addition, the UBR boxes of UBR2, UBR4, and UBR5 have been confirmed to bind type 1 N-degrons. UBR1 and UBR2 can bind type 2 N-degrons via the N domain present. However, UBR4 can also bind type 2 N-degrons, but no clear N domain has been identified^47,48^. The typical UBR box consists of two zinc fingers. The first zinc finger is composed of two zinc ions, each tetrahedrally coordinated but sharing a Cys ligand (Cys127). The second zinc finger is more typical: two zinc coordination residues are located in the linker between the β1 strand and α1 helix (Cys112 and Cys115), another in helix α2 (His133), and the last in the linker between α2 and β2 (His136). All but one of the zinc-coordinating residues are conserved across the UBR family, which suggests that all of the UBR boxes fold similarly^49,50^.

The UBR box is essential for the function of UBR5. For example, in inflammatory response, activated inflammatory caspases or other proteases involved in the immune response produce various proinflammatory fragments, some of which contain destabilizing N-terminal residues, including Asn120-CASP1, Gln81-CASP4, Gln139-CASP5, and Cys149-Rab39 produced by caspase, as well as endopeptidase DPP1. These fragments are substrates of N-recognin, including UBR5 as well as UBR1, UBR2, and UBR4, and are degraded by the UPS. This is evidenced by the significant reduction in LPS-induced IL-1β secretion caused by depletion of UBR5, suggesting the potential function of UBR5 in inflammation^28,51^.

### MLLE/PABC domain

MLLE (previously named poly(A)-binding protein C-terminal domain, PABC) is a peptide-binding domain that exists in PABP (polyadenylate binding protein) and UBR5. The MLLE domain of PABP is a peptide interaction domain consisting of five α-helices arranged as arrows that recruited various regulatory proteins and translation factors to Poly(A) mRNA through binding to a conserved 12-amino acid peptide motif named PAM2 (PABP interaction motif 2)^52,53^. The structure of the human UBR5 MLLE domain is similar to PABP, except that the MLLE domain of UBR5 only consists of four alpha helices, and the structure of the peptide-bound UBR5 MLLE shows a helical bundle with four α-helices folded into a right-handed superhelix^54^. Affinity measurements revealed subtle but clear differences in the peptide specificity of UBR5 compared to PABP, such that the UBR5 MLLE domain has a lower affinity for the PAM2 peptide than the PABP MLLE domain. Two factors may contribute to these differences. The first is the MLLE domain first-order sequence, which may influence and modify peptide-protein intermolecular contacts and thus change the weight of charged versus hydrophobic interactions. The second is the lack of the first N-terminal helix in the UBR5 MLLE domain. In PABP, this helix does not directly contact the bound peptide but may indirectly affect binding through its effect on the α2-helix^36,55–57^.

The MLLE domain of UBR5 mainly makes a difference in the substrate selectivity, which in turn regulates the physiological function of UBR5. For instance, human GW182 is the core component of miRNA-induced silencing complexes, UBR5 is considered a key component of the miRNA silencing pathway, and the MLLE domain is essential for its silencing function. UBR5 regulates miRNA-mediated gene silencing in an E3 ligase-independent manner by targeting the GW182 family of the Argonaute-miRNA complex. UBR5 recruited the translation effectors GW18 and Tob1/2 but did not promote their proteasomal degradation^58^. In addition, the MLLE domain of UBR5 has been shown to bind Paip (poly(A) binding protein interacting protein), a PABP-interacting protein. UBR5 targets Paip2 for ubiquitination and proteasomal degradation when PABP is depleted, and Paip2 binds only to full-length UBR5 or fragments containing MLLE, confirming that the interaction between UBR5 and Paip is direct and specific for the MLLE domain of UBR5. The MLLE domain in UBR5 is located on the N-terminal side of the catalytic HECT domain, separated by only about 50 residues. The N-lobe of the HECT domain contains PAM2-like sequences. MLLE can interact with the N-lobe of the HECT domain in a PAM2-dependent manner, and the PAM2 peptide competes with the N-lobe of HECT for MLLE^36^. In addition, UBR5 was found to promote PD-L1 transcription in triple-negative breast cancer (TNBC) in a PABC-dependent manner without requiring its E3 ligase activity. Mutants of UBR5 PABC completely lost the ability to induce IFN-γ on the PDL1 promoter, indicating the importance of this domain in mediating immune evasion in TNBC^57^.

### UBA domain

The UBA domain was originally discovered in a variety of proteins involved in ubiquitination^59^. The 3D structure of the UBA domain shows a three α-helices bundle that often contains surface patches of hydrophobic residues involved in protein-protein interactions. Most UBA domains can bind Ub or ubiquitin-like domains. The two structures of the UBA domain and the complex with monoubiquitin determined by nuclear magnetic resonance spectroscopy show that the first loop of the UBA domain and the C-terminal portion of α3-helix bind to a pan-β-fold centered on Ile44. In particular, the conserved Met residue of the α1-α2 loop in many UBA domains forms a critical hydrophobic contact with Ub^60–62^. The affinity of UBR5 UBA domain mono-Ub binding is similar to the UBA domain of Cbl-b (casitas B-lineage lymphoma-b) Ub ligase, but it shows a relatively higher affinity for K63-linked Ub chains compared to the Cbl-b UBA domain. However, the UBA domain of UBR5 does not bind significantly more tightly to polyubiquitin chains than to mono-Ub chains. UBR5 lacks the conserved Met residue found in the α1-α2 loop of other UBA domains. Instead, it possesses two additional residues in the α1-α2 loop in which the hydrophobic residue Val (valine) replaces the conserved Met. The large α1-α2 loop is characteristic of the CUE (coupling of Ub conjugation to endoplasmic reticulum degradation) domain, but may also occur in other UBA domains, examples include the domain of the Ubx5 protein^34,63^.

### Other domains

In addition to the domains mentioned above, UBR5 also has several other domains with different functions to help UBR5 fulfill various physiological functions, such as the SBB1. SBB1 contains five β chains with an SH3-like fold and may be involved in substrate binding. UBR5 can assemble a tetramer through an SBB2-SBB2 interaction between the two dimers, and the tetramer architecture orientates all four UBR boxes and four HECT domains toward the interior of the large central compartment, suggesting that it can encapsulate and simultaneously add multiple Ub to the substrate, but the tetramer is less stable than the dimer^32^. There is also a β-propeller domain below SBB1, which together form the NTR of UBR5. The β-propeller domain is a seven-blade with a central hole, each containing four β-strands. β-propellers are also frequently involved in protein-protein interactions. However, the C-terminal HECT and MLLE domains of UBR5 directly interact with substrates, and the central pore of the β-propeller is partially covered by SBB1 at the top. Therefore, whether the NTR (SBB and β-propeller) of UBR5 also contributes to substrate binding or interacts with other protein partners still needs to be further investigated^32,64^. In addition, UBR5 also has two nuclear localization sequences (NLS) that are recognized by armadillo repeats of importin α. The NLS of UBR5 is classified into two types, one bipartite and one simple, and both are required for importin α binding, indicating that UBR5 functions in the nucleus^65^.

**Figure 4:**
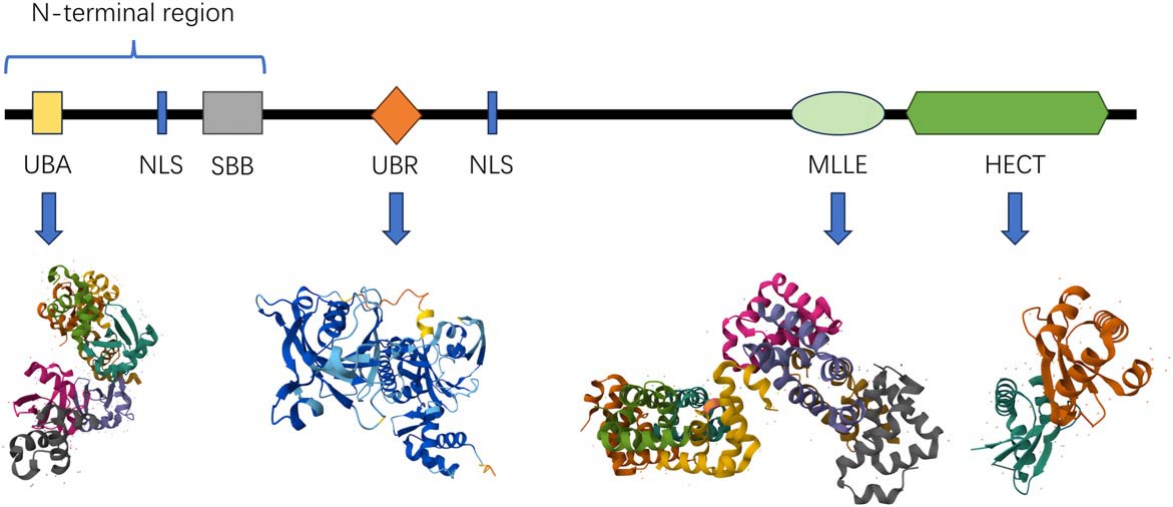
Structural domains and respective crystal structure of UBR5 from N-terminal to C-terminal.

Overall, UBR5 has complex structural domains from N-terminal to C-terminal namely UBA, NTR (SBB and β-propeller), UBR Box, MLLE/PABC domain, and HECT domain, which are indispensable to the function of UBR5 and play various roles in physiological and pathological processes, including immune system regulation, regulation of nuclear hormone receptor stability, maintenance of chondrogenic homeostasis and inhibition of chemotaxis, maintenance of normal DNA replication, as well as in various cancers. In addition to the regulation role in normal physiological processes, the application of UBR5 domains in different cancers has been reported. For instance, UBR5 plays an important role in IFN-γ-induced PD-L1 transcription in TNBC in an E3 ubiquitination activity-independent manner to promote tumor immune evasion, and the MLLE/PABC domain is essential for this effect. The MLLE/PABC domain deletion mutant of UBR5 completely loses the IFN-γ-inducibility on the PD-L1 promoter, suggesting that this domain is pivotal for regulation^57^. Besides, studies have found that in CRC, UBR5 can be recruited by CDK1 (cyclin‐dependent kinase 1) and catalyze ACSL4 (Acyl‐CoA synthetase long‐chain family 4) polyubiquitination through the HECT domain, thereby inhibiting ferroptosis and conferring oxaliplatin resistance to CRC cells^66^. In mantle cell lymphoma (MCL), UBR5 was found with most mutations occurring within the HECT domain. The loss of the UBR5 HECT domain results in blocked B cell maturation in the spleen^41^. Therefore, the potential value of UBR5 for clinical applications in cancer is great. Unfortunately, there is still a lack of research on inhibitors targeting UBR5. At present, the structure of UBR5 has been well studied, and the development of UBR5 inhibitors targeting different structural domains of UBR5 and their application in disease-targeted therapy is promising and deserves further in-depth investigation (Fig. 4).

### UBR5 in immunity

#### UBR5 and immune cell

Some studies have shown that UBR5 deficiency activates naïve CD4+ and CD8+ T effector cells in tumor-draining lymph nodes by inducing the activation of DCs, resulting in the recruitment of effector cells into the tumor, which suppresses tumor growth by inhibiting angiogenesis and inducing tumor cell death^67^. In addition, UBR5 interacts with DUBA, a negative regulator of IL-17 in T cells, to inhibit DUBA abundance in naïve T cells and thereby up-regulate IL-17, functioning to regulate the inflammatory response. Mice with DUBA-deficient T cells develop increased inflammation in the small intestine after an attack with an anti-CD3 antibody^68^. In ovarian cancer (OC), UBR5 enhances OC growth and metastasis through CCL2/CSF-1-mediated immunosuppression of TAM infiltration. UBR5-deficient mice exhibit significantly attenuated tumor growth and reduced peritoneal infiltration of myeloid populations (especially CD11b+F4/80+ macrophages). The chemokines and cytokines CCL2 and CSF-1, which are involved in macrophage recruitment, are important for the recruitment of inflammatory monocytes to the tumor site and their differentiation into TAM. UBR5 directly or indirectly transcriptionally regulates CCL2/CSF-1 via the p53-β-catenin-CCL2 axis, which regulates TAM infiltration. These results suggest that defective macrophage recruitment caused by UBR5 depletion is the primary cause of reduced OC tumor load and prolonged survival^69^. UBR5 also plays a critical role in the maturation and activation of B cells in Mantle-cell lymphoma, particularly in its HECT structural domain. Deletion of the HECT structural domain results in the blockage of maturation of both B cells and follicular B cells, resulting in follicular B cells with aberrant phenotype and decreased GC formation, and fewer antibody-producing plasma cells, suggesting a functional defect^41^.

#### UBR5 and PD-L1 in TME

The role of UBR5 in immunity has been investigated a lot. As we know, tumor cells respond to elevated IFN-γ levels in the tumor microenvironment (TME) by upregulating PD-L1 expression to evade immune surveillance. It has been found that the tumorigenic activity of UBR in TNBC exerts a paracrine effect mainly through its interaction with the adaptive immune apparatus, independently of the adaptive immune system, and even of the E3 Ub ligase activity of UBR5. UBR5 regulates the immunoreactive state of T-lymphocytes. UBR5-deficient tumors have a significantly increased proportion of infiltrating CD8+ T cells and exhibit enhanced granzyme B expression, suggesting that UBR5 promotes tumor growth in a manner dependent on limiting CD8+ T cell activity^70^. Further studies revealed that UBR5 is required for IFN-γ-induced PD-L1 gene expression. The number of CD8+T cells and their cytolytic activity were strongly increased in UBR5-deficient tumor cells. Mechanistically, the JAK-STAT signaling pathway plays an important role in the regulation of IFN-γ-stimulated PD-L1 by UBR5. UBR5 is essential for IFN-γ-mediated transcriptional activation of STAT1 and IRF1. UBR5 silencing resulted in a reduction of IFN-γ-stimulated mRNA and protein levels of both IRF1 and STAT1. These results suggest that upon IFN-γ activation, PDL1 transcription enhanced by UBR5 is mediated through STAT1 and IRF1 and that UBR5-mediated transactivation of PD-L1 is not dependent on its E3 ligase activity but on the PABC structural domain^57^.

### UBR5 in virus

#### RNA virus

It was found that ORF4b inhibits the production of antiviral cytokines in the cytoplasm and nucleus, which may be related to the high lethality of MERS-CoV. UBR5 regulates the stability of ORF4b by acting on the lys36 site of ORF4b to specifically regulate the ubiquitination and degradation of ORF4b. When UBR5 was knocked down, the ability of ORF4b to suppress innate immunity was enhanced and MERS-CoV replication was stronger. These results suggest that UBR5, as an anti-MERS-CoV host protein, targets and degrades ORF4b proteins through the Ub-proteasome system, thereby attenuating the anti-immune capacity of ORF4b and ultimately inhibiting MERS-CoV immune escape^42^. UBR5 is also involved in mediating the cellular TER94/VCP-ZKVI (transitional endoplasmic reticulum 94/valosin-containing protein-Zika virus) capsid interaction. UBR5 is a TER94/VCP cofactor during ZIKV infection. UBR5 directs the interaction of TER94/VCP with capsid and ultimately coordinates the breakdown of nucleocapsid structures, thereby exposing the viral RNA genome to the cytoplasm. Thus, capsid can be degraded by the proteasome complex^71^. HBZ (HTLV-1 basic leucine zipper factor), a protein encoded by human T-cell leukemia virus type 1 (HTLV-1), is the only protein that consistently expressed in adult T-cell leukemia/lymphoma (ATL) tumor cell lines. UBR5 has been shown to positively regulate cell proliferation in T-cell lymphomas by interacting with the central basic region of the HBZ (specifically BR1) and regulating the stability of HBZ proteins, and the UBR5 protein is overexpressed in T-cell lymphoma cell lines. This demonstrates the regulatory potential of UBR5 in T-cell-associated tumors^72^. HIV-1 accessory protein Vpr leads to increased TERT ubiquitination by enhancing the interaction between TERT (telomerase reverse tranase) and the VPRBP substrate receptor of the UBR5-DDB1-VPRBP E3 ligase complex, thus increasing the TERT ubiquitination, downregulating TERT levels and inhibiting telomerase activity, suggesting a possible molecular mechanism for telomerase dysregulation in HIV-1 pathogenesis^73^.

#### DNA virus

MCMV m139 (murine cytomegalovirus 139) is required for efficient viral replication in bone marrow-derived macrophages and endothelial cells in vitro as well as for viral transmission in vivo. UBR5 interacts with m139 to limit MCMV replication. Knockdown of UBR5 in endothelial cells and bone marrow-derived macrophages rescued the replication defects of m139-deficient MCMV^74^. UBR5 has also been found to interact with HBV (Hepatitis B virus) lys7 through the K29 site to form a Ub chain, which may have an important role in HBc (HBV core protein) transport and viral release^74^. The U14 protein encoded by HHV-6 (human herpesvirus-6) induces cell cycle arrest in the G2/M phase by binding to UBR5. U14 co-localizes with UBR5 in the nucleus, which is mainly associated with the C-terminus of U14. However, whether this interaction relationship requires the deubiquitinating enzyme activity of UBR5 and the mechanism of action needs to be further verified^75^. UBR5 has also been identified as an interacting partner of the HPV-18 E6 oncoprotein. Reduced levels of EDD expression enhanced the resistance of HPV-18-positive cells to apoptotic and growth arrest stimuli. This study suggests that changes in UBR5 expression levels can affect the ability of E6 to target various substrates for protein (especially p53) hydrolysis, thereby directly influencing the development of HPV-induced malignancies^73^.

Overall, UBR5 has great potential in immune regulation and is involved in the modulation of immune cells and cytokines as well as viral infections. The regulation of the immune microenvironment by UBR5 is complicated. Whether the role of UBR5 in the immune microenvironment requires its catalytic activity or which structural domain is closely associated with it warrants deeper explorations to refine the knowledge of the functions of UBR5 (Table 1).

**Table 1:** Mechanisms and functions involved in UBR5 in different viral infections.

Virus	Genome	Mechanism	Function	Reference
MERS-Cov	ssRNA	UBR5 mediates the proteasomal degradation of ORF4b by catalyzing the formation of a ubiquitinated chain at the Lys k36 site of ORF4b through its HECT structural domain	Abrogate the ability of ORF4b to suppress the host innate immune system	(Zhou *et al*. 2022)^42^
ZKIV	ssRNA	UBR5 acts as a cofactor for TER94/VCP, mediating the interaction between TER94 and ZIKV capsid, and then translocating the capsid to the proteasome for degradation	Expose the viral RNA genome to the cytoplasm and facilitate viral RNA decapsidation and infection	(Gestuveo *et al*. 2021^71^)
HTLV-1	ssRNA	UBR5 is upregulated in ATL and interacts with the BR1 region of the viral gene HZV, which is encoded by HTLV-1 and essential for viral infectivity or proliferation, thus regulating the stability of HBZ by ubiquitination	Enhances the proliferation of T-Cell Leukemia/Lymphoma cells via the interaction between UBR5 and HBZ	(Panfil *et al*. 2018)^72^
HIV-1	ssRNA	The HIV-1 accessory protein Vpr targets TERT, a catalytic subunit of telomerase, enhancing the interaction between TERT and the VPRBP substrate receptor of the UBR5-DDB1-VPRBP E3 ligase complex, resulting in the increased ubiquitination of TERT and inhibition of telomerase activity	Impair the antiviral activity of T lymphocytes against HIV-1	(Wang *et al*. 2013^176^)
HBV	dsDNA	UBR5 interacts with HBc and catalyzes ubiquitin chain at the k29 site of HBc lys7	Unclear	(Langerová *et al*. 2020)^175^
HHV-6	dsDNA	UBR5 interacts with the HHV-6-encoded U14 protein and induces cell-cycle arrest at G2/M phase	Induce cell-cycle arrest	(Mori *et al*. 2015)^75^
HPV-18	dsDNA	EDD interacts with E6 and E6AP, enhancing the level of E6AP ubiquitination and resulting in negative effect on the capacity of E6 to direct its substrates for degradation The loss of EDD enhances the level of E6/E6AP expression and consequent p53 degradation	EDD has a potent tumor suppressor function in the context of HPV infection, most likely through its capacity to affect the activity of E6	(Tomaic *et al*. 2011)^73^
MCMV	dsDNA	UBR5 and DDX3 interact with m139 that encoded by MCMV and co-localized in viral replication compartments in the cell nucleus to regulate the replication of MCMV	Counteract the host factors UBR5 and DDX3 to subvert host antiviral responses and promote viral replication	(Puhach *et al*. 2020)^74^

#### UBR5 in DDR

Accurate DNA replication is essential for maintaining genome integrity and cell survival. RNF168 plays an important role in DDR and has been meticulously studied. Once recruited to DNA damage sites, RNF168 gradually diffuses from the DNA double-strand break (DSB) to undamaged chromatin. Uncontrolled amplification of chromatin ubiquitination may have deleterious consequences^76^. UBR5 can control the DDR by interacting with and regulating the activity of RNF168 through a variety of pathways. UBR5 has been shown to interact with components of the replication fork, including the translesion synthesis (TLS) polymerase polη. UBR5 downregulation regulates H2A ubiquitination levels through the RNF8-RNF168 axis, leading to elevated ubiquitinated H2A (UbiH2A), which allows polη to be more extensively recruited into chromatin. Since polη is close to the replication fork during the S phase and has unrestricted access to the replication machinery, it can interrupt S phase progression, leading to the accumulation of single-stranded DNA (ssDNA). Silencing of RNF168 can reverse the delay in S phase progression and the accumulation of ssDNA caused by UBR5 knockdown^77^. TRF2 prevents the propagation of the DDR by recruiting MRE11 and UBR5, and blocking the function of RNF168 by ubiquitination. Combined inhibition of MRE11 and UBR5 leads to a significant increase in telomere recruitment and telomere fusion of RNF168 and p53BP1 (p53-binding protein 1) and ultimately to cell death. This effect can be reversed by inhibition of RNF168, suggesting that MRE11 and UBR5 synergistically inhibit dysfunctional telomere fusion^78^. TRF2 can also cut the DNA damage signaling cascade at the level of RNF168, thereby preventing 53BP1 localization and chromosome fusion. This terminal protective effect of TRF2 is mediated by the BRCA1 complex through its associated deubiquitinating enzymes BRCC3 and UBR5^25^. In addition, TRIP12 and UBR5 have been found to cooperatively inhibit RNF168. In the absence of TRIP12 and UBR5, RNF168 accumulates to supraphysiological levels, evading the fine-tuning action of upstream regulators and triggering the diffusion of histone ubiquitination from the DSB site^79^.

ATM kinase is part of the phosphatidylinositol 3-kinase-related kinase (PIKK) family, which activates cell cycle checkpoints and promotes DNA repair in response to DNA damage or replication blockade^80^. Studies have confirmed that the interaction of PPARγ with UBR5 is required for UBR5-mediated ubiquitination of a variety of substrates, including the ATM interactor (ATMIN). In the absence of PPARγ or UBR5, ubiquitination of ATMIN is attenuated and protein levels are elevated. PPARγ promotes UBR5 catalytic activity and regulates ATMIN levels, thereby permitting efficient ATM phosphorylation and initiation of DNA repair in response to DNA damage^81^. In addition, UBR5 was identified as a key component of ATM activation in response to IR (ionizing radiation). UBR5 interacts with ATMIN and catalyzes ubiquitination of ATMIN at the lys238 site in an IR-stimulated manner, thereby reducing ATMIN-ATM interactions and facilitating MRN (MRE11-RAD50-NBS1)-mediated signaling, and thus responding to DNA damage^82^.

Previous studies have reported that UBR5 interacts with the BRCA1 C-terminal (BRCT) domain of TopBP1 (topoisomerase IIβ-binding protein 1). X-ray irradiation may have reduced the ubiquitination of TopBP1 via phosphorylation, resulting in the co-localization of upregulated TopBP1 with the sensing protein γ-H2AX to DNA breaks, which contributes to a nuclear environment that protects DNA terminals and their repair^83^. Recent studies have shown that TopBP1 and PHF8 (plant homeodomain finger protein 8) can act under replication stress to promote the restart of DNA synthesis at stalled forks. UBR5 mediates TopBP1 ubiquitination and degradation through its HECT structural domain, whereas PHF8 binding to TopBP1 protects TopBP1 from degradation by UBR5. PHF8 deficiency destabilizes TopBP1 protein levels through UBR5-mediated TopBP1 ubiquitination and degradation^84^. The above results suggest that UBR5-mediated TopBP1 ubiquitination is important in regulating the DDR.

OTUD5 is a regulator of DNA damage-induced transcriptional repression. UBR5 and OTUD5 can co-localize and interact at DSB sites, stabilizing UBR5 and inducing transcriptional silencing at DSB lesions by controlling the Pol II pathway, inhibiting RNA Pol II elongation and RNA synthesis^85^. Similarly, BMI1 (a key regulatory component of Polycomb Repressive Complex 1) and UBR5 inhibit Pol II elongation and RNA synthesis at UV-induced DNA damage. Mechanistically, UBR5 may inhibit FACT (facilitates chromatin transcription) enrichment and Pol II elongation at lesions by promoting ubiquitination of SPT16^86^.

#### UBR5 in protein quality control

Thirty percent of proteins require further assistance from specialized chaperones during transcription, translation, folding, and assembly of newly synthesized polypeptides into functional three-dimensional structures to obtain their biologically active conformation. Any misfolded or misassembled protein will be susceptible to cytotoxic aggregation, and these defects can result in many diseases such as aging, cancer, and neurodegenerative disorders. UPSs can act as a regulator by selectively degrading incorrectly assembled proteins with potential cytotoxicity to oversee the integrity and quality of the complex process of protein synthesis^87^. For example, an E2 enzyme named UBE2O detects and binds exposed basic and hydrophobic (BH) patches in monomeric α-bead proteins to mediate their ubiquitination, regulating correct α-β dimerization as well as the assembly of hemoglobin complexes^88^. The E3 enzyme HUWE1 can recognize the unassembled ribosomal subunits and ubiquitinate the redundant subunits, while retaining the assembled ribosome, thus functioning as a quality control for ribosomal protein assembly^89^. UBR5 has similar functions in protein quality control. It has been reported that UBR5 and BAG6 chaperone complexes are C9orf72-interacting proteins that lead to the degradation of C9orf72 by forming a Ub chain at the K11/K48 site. The BAG6 chaperone complex binds to C9orf72 after synthesis and ensures the formation of a functional complex with SMCR8. SMCR8 prevents the rapid degradation of C9orf72 by the proteasome and the loss of SMCR8 is an initiator of the degradation of C9orf72. When SMCR8 is absent, C9orf72 exposes the Lys residue to UBR5 to form a Ub chain at the K11/K48 site, leading to the proteasomal degradation of C9orf72^90^. In addition, UBR5 has been found to create a dynamic network of protein interactions that drive gene expression through orphan quality control. UBR5 establishes dynamic interactions between transcriptional regulators, allowing cells to efficiently execute gene expression and to remain receptive to environmental signals. UBR5 contributes to the degradation of the orphans of multiple transcription factors functioning in a network centered on the c-Myc oncoprotein subunits. By eliminating unpaired transcription factors, UBR5 establishes dynamic interactions that allow cells to efficiently execute gene expression while remaining responsive to environmental changes^24^.

In addition to the major physiological functions described above, UBR5 also plays regulatory roles in other areas. It has been reported that UBR5 ubiquitinates MCC (mitotic checkpoint complex) components and stimulates MCC disassembly from APC/C (anaphase-promoting complex/cyclosome), thus participating in the regulation of mitotic checkpoint inactivation^91^. UBR5 is also involved in the degradation of unfolded antibody-heavy chains in endoplasmic reticulum. UBR5 promotes IgG HC K48 site ubiquitination and subsequent PDIA3-mediated HC cleavage, thereby promoting IgG HC proteasomal degradation^92^. In addition, UBR5 is also involved in skeletal muscle mass regulation as well as anabolism, regulation of ribosomal RNA biogenesis in embryonic stem cells, nociception in chronic inflammatory pain, as well as cell cycle and gluconeogenesis^43,93–97^. This shows that UBR5 has a very wide range of physiological functions. It regulates almost all aspects of life processes and is indispensable for normal organismal growth. At the same time, disorders of UBR5 are bound to cause a wide variety of diseases, not only cancer. The following section summarizes the diseases caused by UBR5 disorders.

### UBR5 in diseases

#### In breast cancer

Breast cancer poses a great burden to women worldwide, with TNBC being the most malignant subtype with the worst prognosis^98^. Initially, in mammary ductal carcinoma, mutation detection in tumor samples by denaturing high-performance liquid chromatography (dHPLC) and direct sequencing uncovered that UBR5 was the only gene possessing synonymous mutations^99^. UBR5 and circUBR5 (hsa_circ_0001819) expression is elevated in TNBC, and deletion of UBR5 and circUBR5 suppresses a range of malignant phenotypes both in vivo and ex vivo^67,100^. Knockdown of UBR5 induces aberrant epithelial-mesenchymal transition (EMT) to inhibit metastasis, and also alters the TME resulting in repression of angiogenesis and increased immune cell infiltration^67^. CircUBR5 is a component of TNBC progression through the circUBR5/miR-1179/UBR5 axis^100^. Programmed cell death 1 (PD-1) and its ligand (PD-L1) inhibit antitumor immunity by hijacking T-cell function, and PD-L1 immunotherapy has achieved promising effects in a variety of solid tumors^101^. PD-L1 is elevated at both mRNA and protein levels in TNBC^102^, and UBR5 can tempt IFN-γ-induced PD-L1 expression. The absence of UBR5 boosted the function of effector T cells, making tumor cells more vulnerable to the c-Met specific human chimeric antigen receptor (CAR) T cells killing, and the combination of UBR5 and PD-L1 displayed more potent power. UBR5 promotes PD-L1 transcription through the PABC structural domain in a mode independent of E3 ligase activity. In the JAK1/2-STAT1/3-IRF1 signaling axis, UBR5 impels the enrichment of STAT1 and IRF1 in the PD-L1 promoter region by increasing PKR-mediated transcription of STAT1 and IRF1, rather than inhibiting the degradation of STAT1 and IRF1, which promotes PD-L1 transcription^57^. The latest studies have revealed a role for UBR5 in postoperative tumor cell metastasis. UBR5 relies on its E3 ligase activity to promote postoperative lung metastasis in TNBC, independent of the original cell. Silencing UBR5 promotes apoptosis and increases chemosensitivity by targeting CDC73, a substrate of UBR5. Also, UBR5 regulates p53 expression through CDC73. Promisingly, these results are consistent with human clinical observations, demonstrating that UBR5 is a very potent targeted therapeutic target with great potential for clinical application^103^. UBR5 is a novel MYC Ub ligase that represses MYC protein expression, and this finding is corroborated in Drosophila: absence of hyperplastic discs, the homolog of UBR5 in Drosophila, leads to elevated Drosophila MYC protein (dMYC). UBR5 and MYC are co-amplified in breast cancer to protect against MYC-mediated apoptosis that is independent of p53, with basal-type breast cancer is the subtype with the most pronounced co-amplification^104^. For estrogen receptor positive (ERa+) breast cancer, which account for the majority of breast cancer, tamoxifen (TAM) is a critically indispensable drug throughout all stages of treatment^105^. UBR5 expression is upregulated in ERa+ breast cancer and contributes to TAM resistance to some extent by stabilizing β-catenin, and inhibition of UBR5/β-catenin signaling restores susceptibility to TAM^106^. Interestingly, UBR5 expression was decreased in the etoposide-resistant sublines MCF-7/1E and MCF-7/4E^107^. A subsequent study further found that the tumor suppressor CDC73 antagonized UBR5-driven invasive breast carcinogenesis and metastasis by inhibiting the expression of β-catenin and E-cadherin, and that UBR5 ubiquitylated and degraded non-phosphorylated CDC73 via the Lys^243^, Lys^247^, and Lys^257^ residues^108^. Distant metastasis of tumors is a cardinal menace in TNBC with very limited treatment, and UBR5 is critical for postoperative lung metastasis in TNBC. Knockdown of UBR5 induced by the addition of doxycycline (Dox) suppresses primary tumor growth, impairs proliferation and increases apoptosis of metastatic cancer cells in the lung. UBR5 acts as a catalyst for tumor metastasis through its own Ub ligase activity, independently of the presence of a primary tumor. The latest research has demonstrated that the tumor suppressor parafibromin encoded by cell division cycle 73 (CDC73) is a substrate for proteasomal degradation by UBR5, which functions in a completely opposite way to CDC73 in regulating tumor cell apoptosis, chemotherapy sensitivity, the number of Ki67+ proliferating cancer cells, and metastatic lung nodules. A negative correlation between UBR5 and CDC73 was observed in primary TNBC tissues, and CDC73 quenched the metastatic activity of UBR5. UBR5 relies on CDC73 to control p53 mRNA levels, and apoptosis caused by UBR5 knockdown is greatly dependent on elevated p53 levels^103^. However, Zhao *et al*.^109^ demonstrated that OTU domain-containing protein 6A (OTUD6A) promotes breast cancer development and resistance to chemotherapeutic agents, whereas UBR5 and OTUD6A antagonize each other, and OTUD6A blocks the interaction of UBR5 with TopBP1 thereby contributing to the progression of cancer.

#### In CRC

CRC is the third most prevalent cancer in the world, resulting in 900 000 deaths per year^110^, and UBR5 plays an indispensable role in the development of CRC. In CRC tissues, UBR5 expression is upregulated at both the mRNA and protein levels, and high mRNA expression of UBR5 represents a lower survival rate^111–113^. The protein of UBR5 is predominantly localized in the nucleoplasm, and high protein expression is associated with a poor prognosis in patients with stage II or III CRC^112^. Meanwhile, in early T-stage CRC patients, the distribution of UBR5 differed significantly between the tumor mutational burdens (TMB)-Low and TMB-High groups, and UBR5 was a high-frequency mutant gene in The Cancer Genome Atlas (TCGA) hypermutated specimens^114^. UBR5 significantly facilitated proliferation, invasion, cell cycle progression, and inhibited apoptosis in CRC cells^111,113,115^. Esophageal cancer-related gene 4 (ECRG4) is a previously reported CRC tumor suppressor gene^116^, and UBR5 was able to directly ubiquitinate ECRG4 to reduce its stability^111^. Proline-rich tyrosine kinase 2 (PYK2) is a tyrosine kinase and UBR5 interacts with PYK2 to regulate mitochondrial oxidative phosphorylation (OXPHOS). Silencing of UBR5 reduces the level of PYK2 to inhibit the process of oxidative phosphorylation thereby suppressing the malignant phenotype of CRC^115^. Oxaliplatin is a first-line drug for the treatment of CRC, and cyclin‐dependent kinase 1 (CDK1) promotes oxaliplatin resistance mainly by degrading Acyl‐CoA synthetase long‐chain family 4 (ACSL4) via UBR5 ubiquitination thereby inhibiting ACSL4-mediated lipid peroxidation and ferroptosis processes^66^.

#### In OC

Excluding cervical cancer, OC is the second leading cause of gynecological cancer death among women worldwide, with a 5-year survival rate of only 47%^117^. UBR5 is an essential prognostic indicator in serous epithelial OC and is indispensable for cancer progression^118^. UBR5 is highly expressed in OC tissues, and the deficiency of UBR5 impairs multiple cancer-related properties: depletion of UBR5 impairs epithelial properties and attenuates ID8 tumor colonization of the lungs; affects the immunosuppressive properties of TME and reduces the recruitment of macrophages in a paracrine manner in the TME; cytokine/chemokine secretion is compromised, with the paracrine factors CCL2, M-CSF and β-catenin all downregulated^69^. Cisplatin is a conventional drug for OC treatment, and overexpression of UBR5 facilitates the development of cisplatin resistance^69,118–120^, with Ub ligase activity of UBR5 being imperative for cisplatin resistance^120^. UBR5 stimulates the transcription of myeloid cell leukemia sequence 1 (Mcl-1) in a manner that is independent of Ub ligase activity, and upregulation of Mcl-1 protects cells from apoptosis induced by UBR5 knockdown^120^. UBR5 interacts with modulator of apoptosis protein 1 (MOAP-1), ubiquitinates MOAP-1 at the S-G2 phase specifically, and also synergistically regulates MOAP-1 stability with other components of the E3 ligase EDVP complex. MOAP-1 is a regulator of Bax and silencing of UBR5 allows MOAP-1 to accumulate thereby increasing Bax activation resulting in apoptosis^119^. UBR5 is positively correlated with the expression of the oncogene Golgi phosphoprotein 3 (GOLPH3), which may regulate UBR5 through the GOLPH3–Wnt/β‐catenin–EMT axis^121^. One recurrent mutation in UBR5 was identified in each of targeted (c.935A>C; p.E312A) and whole genome sequencing (c.G953A; p.R318H) performed on low-grade serous ovarian carcinoma (LGSOC), the pathological significance of which remains to be investigated^122^. In the high-grade ovarian serous carcinoma (HGOSC) cohort, UBR5 is identified as a highly correlated gene among the Tier 1 Cancer Gene Census in the Catalog of Somatic Mutations in Cancer (CGC-COSMIC)^123^.

#### In HCC

HCC is the most common cancer in the world and its incidence and mortality rates have been increasing in recent years^124^. The antioncogene C2orf40, also referred to as ECRG4, is further identified as a potential therapeutic target in HCC on the basis of previous studies in CRC^111^. C2orf40 is down-regulated due to promoter hypermethylation in HCC, and C2orf40 interacts with UBR5 to regulate the p21 expression via the C2orf40/UBR5/p21 axis^125^. UBR5 is upregulated in HCC tissues^126,127^ and negatively correlates with tumor prognosis^127^. UBR5 interacts with AXIN1, which regulates β-catenin, and knockdown of UBR5 can inhibit ubiquitination of AXIN1^126^. High expression of YWHAZ in HCC tissues rescues cell proliferation inhibition induced by UBR5 silencing^127^. Echinacoside (ECH), extracted from the Chinese herb Cistanche salsa, suppresses the oncogenic effects of UBR5 and could be delivered to HepG2 cells by a carrier consisting of mesoporous silica nanoparticles (MSN), galactose (GAL), and poly ethylene glycol diglycidyl ether (PEGDE) (ECH@Au@MSN-PEGDE-GAL, or ECH@AMPG), which significantly injured the aerobic glycolysis of HCC cells and fostered cell apoptosis^126^.

#### In MCL

MCL is a B-cell non-Hodgkin’s lymphoma (NHL) with a very poor prognosis, characterized by the translocation (11;14) (q13;q32) and overexpression of cyclin D1^128^. Barbara Meissner and her team conducted RNAseq on 18 patients and 2 MCL cell lines, further focusing on 18 genes based on the preliminary results, with a total of 102 MCL tumors analyzed for sequence^129^. Sequencing results revealed that in addition to known somatic mutations in ATM, TP53, NOTCH, and CCND1, UBR5, which had never been mentioned before, was discovered for the first time to be mutated in 18% (8/102) of MCL, which makes UBR5 the third most commonly mutated gene in MCL^129^. Subsequently, cDNA sequencing was performed on all samples and finally 11 deleterious mutations and 7 missense mutations in UBR5 were identified in 18 cases^129^. All mutations in UBR5 are clustered within the HECT structural domain, a disease-specific genetic feature of MCL, which may account for the deletion of conserved cysteine residues^41,129^. After that, Swenson *et al*.^41^ constructed a UBR5 HECT domain mutant mouse model and demonstrated that deletion of the HECT domain resulted in compromised B-cell maturation, as well as altering the phenotype and cell cycle of mature splenocytes. Six of the 18 mutations (33%) in UBR5 involve splice sites, and B cells with mutations in the HECT domain have increased levels of proteins associated with mRNA splicing, and the use of mass spectrometry to explore proteins interacting with UBR5 reveals that they can be labeled as spliceosome core components^41,129^. Deletion of the HECT domain enhances the stability of UBR5 and the U5 small nuclear ribonucleoprotein (snRNP) complex, which interacts with UBR5, and high expression of UBR5 and the components of the U5 snRNP complex (EFTUD2, SNRNP200, PRPF8, and DHX15) is a hallmark of MCL^41^.

#### In glioma

Gliomas are the most common primary brain tumors and glioblastomas (GBM) are the most common malignant form in adults^130^. UBR5 participates in the regulation of tumor progression by functioning through the miR-361-5p/UBR5/ATMIN axis: UBR5 is upregulated in glioma tissues, and UBR5 levels are positively correlated with tumor progression and negatively correlated with survival^131,132^; miR-361-5p and ATMIN are down-regulated, and miR-361-5p targets UBR5, and UBR5 deletion rescues deterioration caused by down-regulation of miR-361-5p in tumor progression^131^. UBR5 interacts with and ubiquitinates ATMIN, and inhibition of UBR5 reduces the growth of glioma cells as well as the acquisition of a malignant phenotype^131^. UBR5 promotes EMT and also regulates the ECRG4/NF-kB signaling pathway^132^.

#### In nasopharyngeal carcinoma (NPC)

NPC originates from the epithelial tissue of the nasopharynx, is relatively rare, and has a highly unbalanced geographic distribution^133^. A large number of gene rearrangements in NPC were identified by paired-end whole-transcriptome sequencing, with novel fusion genes arising from UBR5 on chromosome 8q22.3 and zinc finger protein 423 (ZNF423) on chromosome 16q12.1 found in 12/144 (8.3%) primary tumors^134^. The full length of the UBR5-ZNF423 fusion gene is 1031 bp, including the 5′-UTR and exon 1 of UBR5 and exons 7-9 of ZNF423, and the product contains 94 amino acids with a predicted molecular weight of 10.8 kDa, and only two amino acids at the N-terminal end of the protein are encoded by UBR5^134^. The UBR5-ZNF423 fusion protein is a novel carcinogen and potential therapeutic target, whose expression promotes NPC cell growth and may drive the transformation of some NPC by altering the activity of EBFs (Early B-cell factors)^134^. CircMAN1A2 is highly expressed in NPC tissues, promotes the malignant phenotype and EMT of NPC, and acts as a sponge for miR-135a-3p to inhibit the expression of miR-135a-3p^135^, which targets and inhibits UBR5. Comparable to downstream mechanisms in gliomas, UBR5 interacts with and ubiquitinates ATMIN, which can inhibit malignant growth and EMT in NPC^135^.

#### In prostate cancer (PC)

PC is the most common cancer among men, with genetic mutations thought to be the main driver, and the risk of developing cancer increases with age^136^. Androgen-deprivation therapy usually achieves good feedback as a first-line treatment for PC, but these patients invariably end up relapsing and progressing to hormone-refractory prostate cancer (HRPC)^137^. Docetaxel is a chemotherapeutic agent against HRPC, and UBR5 is upregulated in docetaxel-resistant HRPC cells and clinical samples, and deletion of UBR5 restores cellular sensitivity to docetaxel^138^. UBR5 may be acquired through activation of the Wnt/β-Catenin signaling pathway for docetaxel resistance^138^. Yan *et al*.^139^ also employed two massive clinical omics databases: the cBioPortal for Cancer Genomics and the Prostate Cancer Transcriptome Atlas (PCTA) to conclude that UBR5 is the most common genetic alteration in metastatic castration-resistant prostate cancer (mCRPC), and that UBR5 is positively correlated with tumor progression and aggressiveness. Via ingenuity pathway analysis, UBR5 was observed to be directly associated with four upregulated differentially expressed proteins: FANCD2, XRCC3, AQR, and BUD23, and experimental validation confirmed that UBR5 was negatively correlated with the expression levels of XRCC3 or FANCD2 and could confer sensitivity to Olaparib^139^.

#### In pancreatic cancer

Pancreatic cancer is the fourth leading cause of cancer-related deaths globally, and despite significant advances in research on pancreatic cancer in recent years, the 5-year survival rate for pancreatic cancer is still only 4%^140^. UBR5 expression is upregulated in pancreatic cancer at both mRNA and protein levels, and overexpression of UBR5 is significantly correlated with tumor TNM stage and histological grading, and is an independent prognostic factor for pancreatic cancer^141,142^. Overexpression of UBR5 notably promoted cancer cell growth, migration, and invasion in vitro and in vivo^141,142^. UBR5 interacts with F-actin-capping protein subunit alpha-1 (CAPZA1), and promotes pancreatic cancer progression by downregulating CAPZA1 via the Ub-proteasome pathway which induces actin filament (F-actin) accumulation^141^. FBP1 is a negative regulator of pancreatic cancer, and UBR5 is a capable of triggering aerobic glycolysis in cancer cells by down-regulating FBP1, but interestingly UBR5 does not interact with FBP1. It was illustrated that UBR5 binds directly to C/EBPα, and UBR5 mediates the Lys48 polyubiquitination of C/EBPα to destabilize C/EBPα and hence regulate the transcription of FBP1, and the silencing of UBR5 dramatically increased the protein expression of both C/EBPα and FBP1^142^.

#### In esophageal cancer

Esophageal cancer is a malignant disease with a high mortality rate and a 5-year survival rate of <20%, with two primary histological types: squamous and adenocarcinoma^143,144^. The sex determining region (SRY)-box 2 (SOX2) is an embryonic stem cell transcription factor that is closely associated with a variety of malignancies. The level of SOX2 in esophageal squamous cell carcinoma is weighed by UBR5 and Akt: UBR5 can degrade SOX2 by polyubiquitylation at the K115 site, whereas phosphorylation of the T116 site by AKT protects SOX2 by preventing the binding of UBR5 to SOX2^145^. Luteolin (Lut), a naturally occurring flavonoid, destabilizes SOX2 via the PI3K/AKT pathway consequently inhibiting stemness in paclitaxel (PTX)-resistant cancer cells^146^. UBR5 is down-regulated in PTX-resistant esophageal cancer cells, which may lead to a reduction in SOX2 degradation and hence promote proliferation and stemness of cancer cells^146^.

#### In other cancers

**Figure 5:**
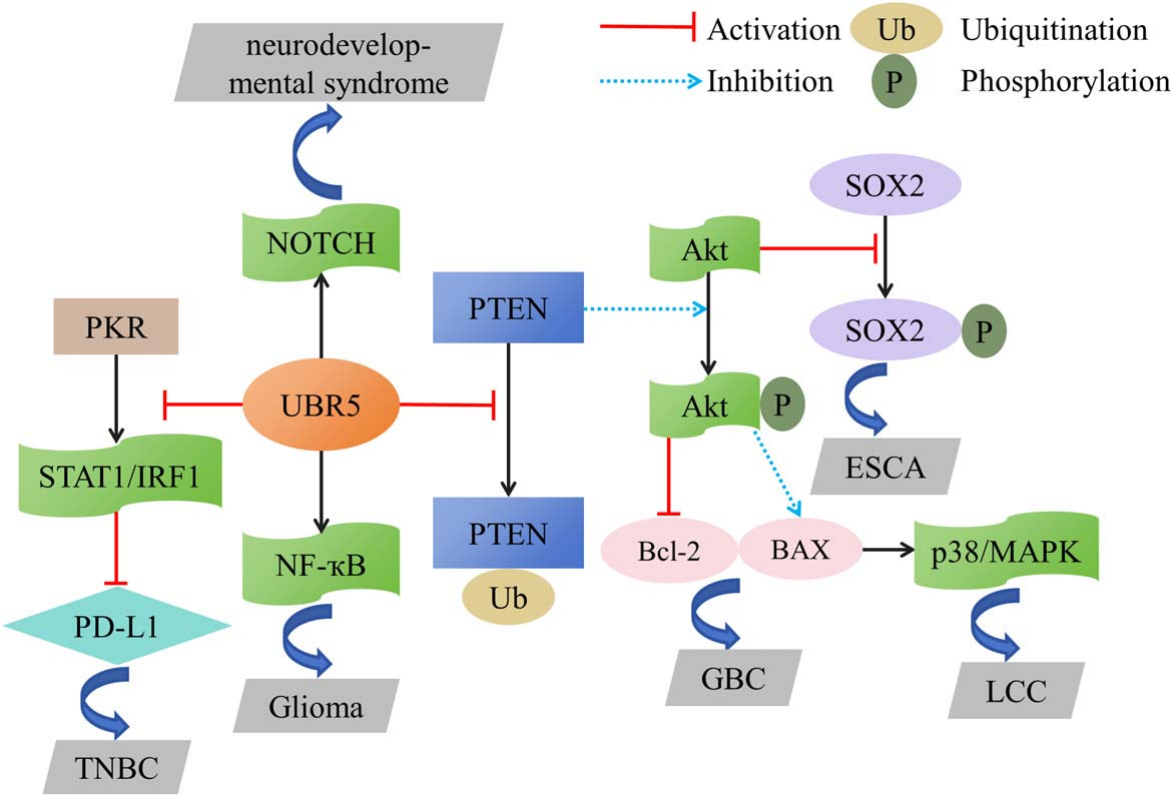
Signaling pathways involved in UBR5 regulates the process of several cancers and diseases.

Lung cancer has long been the most high-profile cancer, claiming more lives than any other cancer^147^. UBR5 is highly expressed and/or mutated in lung adenocarcinomas, and deficiency in UBR5 renders cell growth and clone formation impaired^27^. Using MS and co-immunoprecipitation technology, it was established that UBR5 interacts with proteins of the mTOR complex, DNAPK (DNA damage pathways), GCN1 (a translational activator), CDK1 (cell cycle regulator), and AKT^27^. UBR5 is upregulated in nonsmall cell lung cancer, and UBR5 absence prominently inhibits cell viability in a radiation dose-dependent manner, and induces apoptosis and confers cell radiosensitivity by regulating the PI3K/AKT pathway^148^. Malignant melanoma (MM) is one of the most lethal cutaneous malignancies, with a moderate prognosis before metastasis occurs and a sharp turnaround after metastasis^149^. CSN6, a subunit of the constitutive photomorphogenic 9 (COP9) signalosome (CSN) complex, is a poor prognostic indicator for MM, and UBR5 is a top-down actor in the CSN6-UBR5-cyclin-dependent kinase9 (CDK9) axis, where CSN6 stabilizes CDK9 by down-regulating UBR5 so as to promote MM progression^150^. UBR5 is overexpressed in a diverse range of cancers, such as laryngeal carcinoma (LCC), gallbladder cancer (GBC), and gastric cancer (GC), which notably potentiate the proliferation and invasion of cancer cells in vivo and in vitro^151–154^. In LCC, UBR5 regulates the proliferation and radiosensitivity of LCC cells through the p38/mitogen-activated protein kinase (MAPK) pathway^151^. Although the most common tumor of the biliary system, GBC is relatively uncommon and is often at an advanced stage at the time of diagnosis, with a very high mortality rate^155^. In GBC, UBR5 may degrade phosphatase and tensin homolog deleted on chromosome 10 (PTEN) through the ubiquitination pathway, which is a well-known antioncogene, and PTEN can inhibit the phosphorylation of Akt. Meanwhile, increased phosphorylated Akt can induce BAX accumulation and Bcl-2 depletion, so as to promote GBC progression^153^. In GC, UBR5 interacts with gastrokine 1 (GKN1), a specific protein in stomach, and increases its level of ubiquitination^154^, and UBR5 is also one of the genes with the highest number of copy number variations^156^. UBR5 is also a risk factor for osteosarcoma (OS), and patients with high UBR5 expression may have an immunosuppressive microenvironment^157^. In hypothalamic hamartoma (HH), a case of UBR5 somatic missense mutation was identified, but the implications are still ambiguous^158^. The down-regulation of UBR5 in clear cell renal cell carcinoma (ccRCC) is particularly striking compared to its up-regulation in the vast majority of cancers. UBR5 expression was negatively correlated with tumor stage, sunitinib resistance, and CD163, and combining UBR5 with CD163^+^ tumor-associated macrophages (TAMs) was capable of predicting the prognosis of ccRCC patients more accurately^159^. In papillomavirus (HPV)-driven cervical cancer, UBR5 interacts with the negative tumor regulator tat-interacting protein of 60 kDa (TIP60), which is destabilized by UBR5 via the proteasome pathway^160^. However, another article pointed out that UBR5 binds tightly to HPV-18 E6 and can affect the ability of E6 to direct p53 degradation in vivo, with a positive correlation between UBR5 and p53, and that UBR5 is a powerful tumor suppressor protein in HPV-18-positive cervical cancer^73^ (Fig. 5).

#### Other diseases

Neuropathic pain is usually due to a neurological lesion or disease and often manifests as allodynia and hyperalgesia^161^. ciRNA-Fmn1 is highly conserved in mammals and is primarily expressed in dorsal horn neurons. In chronic constriction injury mice, ciRNA-Fmn1 negatively regulates albumin (ALB), and downregulation of ciRNA-Fmn1 causes upregulation of ALB, leading to neuropathic pain. UBR5 interacts with ALB, and downregulation of ciRNA-Fmn1 reduces UBR5-induced ubiquitination of ALB in the ipsilateral dorsal horn^162^. CircRNA-Filip11, whose expression is increased in chronic inflammation pain conditions, modulates nociception by positively targeting UBR5, with the involvement of miRNA-1224 and Argonaute-2 (Ago2)^96^.

Pandemics of respiratory diseases caused by coronaviruses (CoVs) pose a significant health threat and economic burden, and there is still no effective treatment for them^163^. In Middle East respiratory syndrome (MERS), ORF4b is an auxiliary protein of MERS-CoV that plays a critical role in pathogenesis. UBR5 acts as an anti-MERS-CoV factor in vivo by mediating ORF4b ubiquitination in the cytoplasm and nucleoplasm through the highly conserved K36 lysine residue site resulting in reduced intracellular levels of ORF4b, which allows for enhanced host immunity^42^.

Human herpesviruses (HHV) are a broad category of enveloped, double-stranded DNA viruses that cause lifelong latency after infection^164^. The U14 protein encoded by human herpesvirus-6 (HHV-6) relies on the C-terminus to physically bind to UBR5, and the binding of UBR5 and U14 induces cell cycle arrest in the G2/M phase^75^. Infection with human cytomegalovirus (HCMV) is very common, antibodies can be detected in almost all adults, and when the immune system is deficient it can give rise to serious somatic diseases^165^. HCMV protein synthesis is promoted by polyadenylate-binding protein PABPC1 (PABP1), while UBR5 and Paip2 are PABP1 deterrent proteins, and surprisingly UBR5, Paip2, and PABP1 are increased together after HCMV infection^166^. In mice, UBR5 and DDX3 are replication restriction factors for murine cytomegalovirus (MCMV) and m139 counteracts this inhibition^74^.

The advent of high-throughput sequencing technology has certainly contributed greatly to exploring the underlying pathogenesis of disease. De novo variants in UBR5 were identified in the cohort of patients tested with both developmental epileptic encephalopathies (DEEs)^167^ and Williams-Beuren syndrome (WBS)^168^. After whole-exome sequencing in a patient with very early onset inflammatory bowel disease (VEOIBD), a double allele compound heterozygous nonsynonymous variant of UBR5 was noted, which binds to tetratricopeptide repeat domain 7A (TTC7A), a deficiency of which can be associated with severe intestinal disease^169^. In a neurodevelopmental syndrome with epilepsy, ptosis, and hypothyroidism, UBR5 and UBR7 synergistically regulate Notch signaling^170^.

Huntington’s disease (HD) is an autosomal dominantly inherited neurodegenerative disorder in which abnormal amplification of the CAG sequence in the huntingtin (HTT) gene results in the production of mutant HTT protein (mHTT)^171^. UBR5 degrades HTT proteins, whether normal or abnormal, and reduction of UBR5 can evoke mHTT aggregation and exacerbate neurotoxicity in invertebrate models. Induced pluripotent stem cells (iPSC) can restrain HTT aggregation, and UBR5 is intrinsically enhanced in iPSC, mediating the overall proteostasis of iPSC^172^.

The occurrence of psoriasis adversely affects all aspects of the patient’s body and psyche, and keratinocytes (KCs) can produce miR-381-3p-containing small extracellular vesicles (sEVs) to transfer miR-381-3p to CD4+T cells, and miR-381-3p can target the 3’ untranslated region of UBR5, which induces the polarization of Th1 and Th17 cells to promote the development of psoriasis^173^. The transcriptional activity of RORγt can be increased by the deletion of ras homolog gene family H (RhoH), and the protein level can be stabilized by miR-381-3p, which increases the number of Th17 cells and promotes Th17 cell differentiation, whereas UBR5 can destabilize RORγt to inhibit Th17 cell polarization^173,174^ (Table 2).

**Table 2:** Substrates and mechanisms associated with UBR5 in multiple cancers and diseases.

Diseases	Relevant molecule	Involved mechanism	Reference
Breast cancer	β-catenin	UBR5 contributes to TAM resistance partly through β-catenin signaling	Yang *et al*., 2020^106^
	MYC	UBR5 is a novel ubiquitin ligase for MYC to protect cells from MYC-induced apoptosis	Qiao *et al*., 2020^104^
	miR-1179	UBR5 promotes breast cancer progression through the circUBR5/miR-1179/UBR5 axis	Gong *et al*., 2022^100^
	PD-L1	UBR5 enhances PD-L1 transcription by up-regulating PKR, STAT1 and IRF1 via PABC structural domains	Wu *et al*., 2022
	OTUD6A	OTUD6A blocks the interaction of UBR5 and TopBP1 to promote tumor cell resistance to radiotherapy	Zhao *et al*., 2022
	CDC73	UBR5 facilitates breast cancer development and metastasis by degrading the tumor suppressor CDC73 via the ubiquitination pathway	Xiang *et al*., 2022^108^
		UBR5 relies on CDC73 to control p53 mRNA levels	Yu *et al*., 2023^103^
Cervical cancer	E6	UBR5 binds to HPV-18 E6 to direct p53 degradation	Tomaic *et al*., 2011^73^
	TIP60	UBR5 destabilizes the negative tumor regulator TIP60	Subbaiah *et al*., 2016^160^
Colorectal cancer	ECRG4	UBR5 destabilizes the anti-oncogene ECRG4	Wang *et al*., 2017^111^
	PYK2	UBR5 interacts with PYK2 to regulate mitochondrial oxidative phosphorylation	Qin *et al*., 2023^115^
	ACSL4	UBR5 inhibits ACSL4-mediated lipid peroxidation and iron death processes promoting oxaliplatin resistance	Zeng *et al*., 2023^66^
Esophageal cancer	SOX2	AKT prevents UBR5 binding to SOX2 to promote esophageal cancer progression	Wang *et al*., 2019^145^
Gallbladder cancer	PTEN	UBR5 may degrade PTEN via the ubiquitination pathway	Zhang *et al*., 2019^153^
Gastric cancer	GNK1	UBR5 ubiquitinates GNK1	Yang *et al*., 2016^154^
Glioma	ATMIN	UBR5 participates in the regulation of tumor progression through the miR-361-5p/UBR5/ATMIN axis	Jia *et al*., 2021^131^
	ECRG4	UBR5 promotes glimoa progression by the ECRG4/NF-ҡB pathway	Wu *et al*., 2022
Hepatocellular carcinoma	ECRG4	ECRG4 interacts with UBR5 to regulate p21	Wu *et al*., 2021^125^
	AXIN1	UBR5 ubiquitinates AXIN1	Wang *et al*., 2022
	YWHAZ	The expression of YWHAZ rescues cell proliferation inhibition induced by UBR5 silencing	Huo *et al*., 2022^127^
	ECH	ECH can suppress the oncogenic effects of UBR5	Wang *et al*., 2022
Human herpesviruses	PABP1	UBR5 is a PABP1 deterrent protein preventing HCMV protein synthesis	McKinney *et al*., 2013^166^
	U14	The binding of UBR5 and U14 induces cell cycle arrest in the G2/M phase	Mori *et al*., 2015^75^
	m139	m139 antagonizes UBR5-dependent functions related to RNA metabolism in endothelial cells	Puhach *et al*., 2020^74^
Huntington’s disease	HTT	UBR5 can degrade either normal or abnormal HTT proteins	Koyuncu *et al*., 2018^172^
Melanoma	CSN6	CSN6 downregulates UBR5 to promote melanoma progression	Zhang *et al*., 2021^150^
MERS-CoV	ORF4b	UBR5 acts as an anti-MERS-CoV factor in vivo by ubiquitination degradation of ORF4b	Zhou *et al*., 2022^42^
Neuropathic pain	circRNA-Filip11	CircRNA-Filip11 modulates nociception by positively targeting UBR5	Pan *et al*., 2019^96^
	ALB	Down-regulation of ciRNA-Fmn1 reduces UBR5-induced ubiquitination of ALB in the ipsilateral dorsal horn leading to neuropathic pain	Liu *et al*., 2023^162^
Nasopharyngeal carcinoma	ZNF423	UBR5 fusion with ZNF423 gene produces novel carcinogen	Chung *et al*., 2013^134^
	miR-135a-3p	The circMAN1A2/miR-135a-3p/UBR5 axis can regulate the ubiquitination of ATMIN, like glioma	Dang *et al*., 2023^135^
Ovarian cancer	Mcl-1	Promotion of Mcl-1 transcription by UBR5 reduces apoptosis	Bradley *et al*., 2014^120^
	MOAP-1	Deletion of UBR5 reduces ubiquitination of MOAP-1 increasing Bax activation which leads to apoptosis	Sanchez *et al*., 2016^86^
	GOLPH3	GOLPH3 may regulate UBR5 through the GOLPH3–Wnt/β‐catenin–EMT axis	Sun at al., 2017^121^
Pancreatic cancer	CAPZA1	UBR5 promotes pancreatic cancer progression by downregulating CAPZA1 via the ubiquitin-proteasome pathway which induces F-actin accumulation	Li *et al*., 2021^141^
	C/EBPα	UBR5 interacts with C/EBPα to regulate FBP1 transcription thereby affecting aerobic glycolysis in cancer cells	Chen *et al*., 2021^142^
Psoriasis	RORγt	UBR5 can destabilize RORγt to inhibit Th17 cell polarization	Tamehiro *et al*., 2019^174^
	miR-381-3p	miR-381-3p can target the 3’ untranslated region of UBR5	Jiang *et al*., 2021^173^

Not applicable.

## Conclusion and future perspectives

As a member of HECT E3 ligases, UBR5 has been researched in abundance from structure to multiple biological functions. Studies show that UBR5 has a complex structure to conduct its various functions. The UBR box and HECT domain are the central domains that recognize the N-degrons of substrates and catalyze the ubiquitination separately, which is fundamental for its role as an E3 ligase. The MLLE domain is essential for recognizing the specificity of substrates and UBA domain is responsible for binding Ub. Other domains like SBB and β-propeller also contribute to substrate binding or selectivity. These domains make UBR5 an important regulator in numerous aspects, including DDR, virus infection, protein quality control, and tremendous diseases. Since the structure of protein is the basis of its functions, the development of inhibitors targeting specific structural domains of proteins probably can influence numerous pathophysiology processes and hold great potential for the treatments of relative diseases. The diverse functions of UBR5 are closely related to its various structural domains, and the structure of UBR5 is increasingly being studied, so it is necessary to develop inhibitors based on the linkage between UBR5 structure and functions to generate novel therapeutic strategies. It has been verified that UBR5 can play suppressor or promotor role in different cancers, which depends on the specificity of cells or substrates. Besides, several signaling pathways participates in the pathophysiological process influenced by UBR5, including PI3K/Akt, Notch, MAPK, NF-κB signaling pathways. In summary, the UBR5 is suggested to be an essential regulator in various biological activities, and deeper researches or new therapeutic strategies that targeting UBR5 deserve expectation.

However, some limitations still exist. Most studies focus on the underlying mechanisms of UBR5 biological activities, but pharmaceutical researches targeting the UBR5 inhibitors are still insufficient. This is a key knowledge gap in transferring the basic researches of UBR5 to clinical application and should be a priority for further research. On the other hand, due to the complicate structure of UBR5, whether the function of UBR5 requires its E3 ligase catalytic activity and which domain is needed deserves further investigation. Refinement of these disadvantages may contribute to the development of targeting UBR5 to treat disease.

## Ethical approval

Not applicable.

## Consent

Consent for publication: The authors declare their agreement to public.

Consent to participate: The authors declare their agreement to participate.

## Sources of funding

This study was supported by the Major Program of the National Natural Science Foundation of China (62227803), the National Natural Science Foundation of China (62141109), the Foreword Leading Technology Fundamental Research Project of Jiangsu (BK20212012), and Jiangsu Province Social Development Project (BE2022812).

## Author contribution

Y.L.S. proposed and conceived the project; F.L.Z. and X.H.L.: performed the literature search; Y.Z.W. and K.Y.N.: wrote the original manuscript; Y.L.S.: modified and revised the manuscript; Y.X.L.: drew all the figures; T.Y.C.: edited the tables. All authors improved the manuscript and approved the submission.

## Conflicts of interest disclosure

The authors declares no competing interests.

## Research registration unique identifying number (UIN)

1. Name of the registry: not applicable.
2. Unique identifying number or registration ID: not applicable.
3. Hyperlink to your specific registration (must be publicly accessible and will be checked): not applicable.

## Guarantor

Yewei Zhang.

## Data availability statement

Not applicable.

## Provenance and peer review

Not applicable.
